# Supplementary material for: Regional and cell specific bioactivity of injectable extracellular matrix biomaterials in myocardial infarction
Source: Nat Commun. 2025 Nov 24;16:10387. doi: 10.1038/s41467-025-65351-5 (PMC12644877; doi:10.1038/s41467-025-65351-5)
Supplement: Supplementary file 1 — Supplementary Information [file 41467_2025_65351_MOESM1_ESM.pdf]

Supplementary Information

Regional and Cell Specific Bioactivity of Injectable Extracellular Matrix Biomaterials in Myocardial Infarction

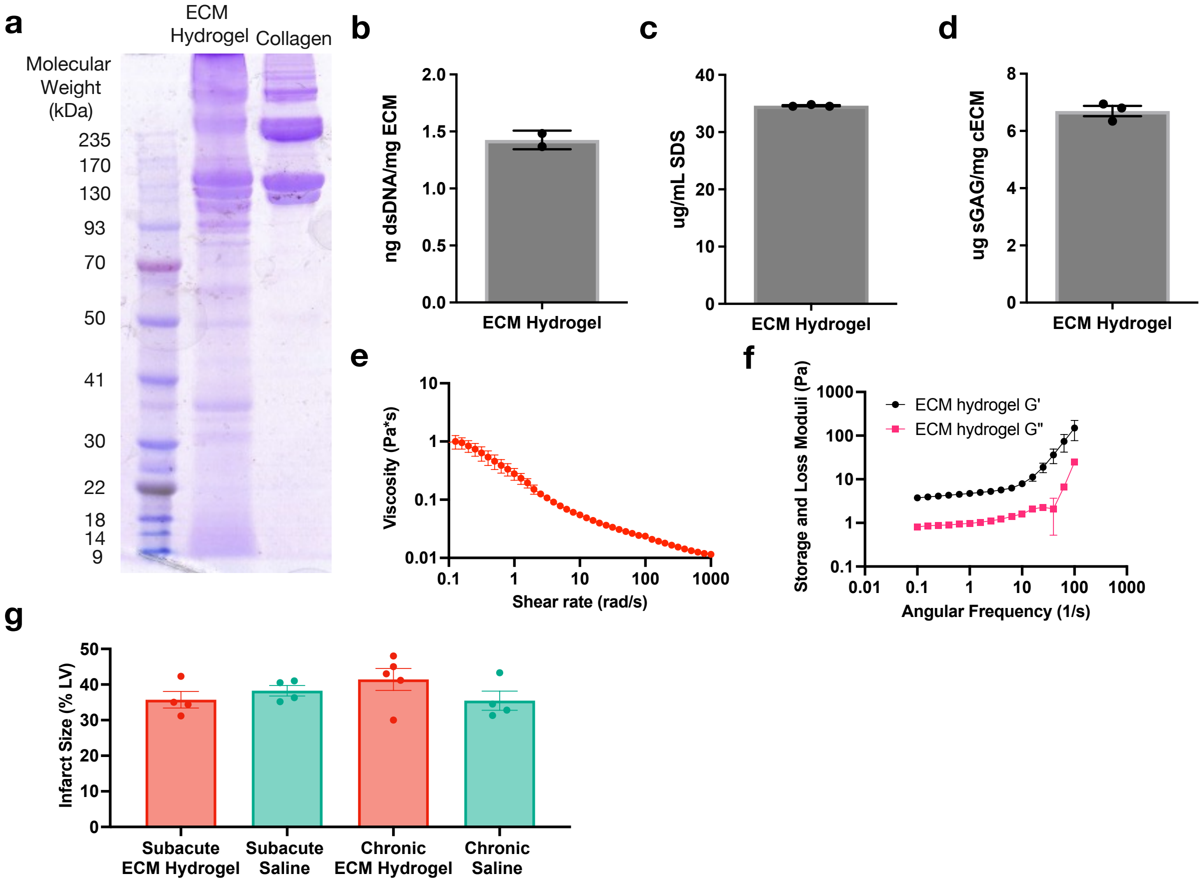

Supplementary Information Figure 1. Quality control for ECM and biological samples. **(a-d)** ECM hydrogel quality control metrics are done to ensure that the ECM hydrogel is similar with each batch. **(a)** Gel electrophoresis exhibits a different protein composition compared to collagen. The experiment was repeated 3 times with similar results. **(b)** Double stranded DNA is quantified to ensure the ECM contains minimal DNA from cellular debris, n = 2 ECM aliquots. **(c)** Sodium dodecyl sulfate (SDS) is quantified to ensure there is no detergent remaining from the decellularization process, n = 3 ECM aliquots. **(d)** Glycosoaminoglycan (GAG) content is quantified to ensure similar GAG concentration across batches, n = 3 ECM aliquots. **(e)** Rheometry is quantified to ensure similar biomechanical properties between ECM hydrogel

batches, n = 3 ECM aliquots. **(f)** The storage (G') and loss (G'') moduli were also calculated for ECM hydrogel batches, n = 3 ECM aliquots. **(g)** Infarct quantification of each sample, split by condition and MI model, demonstrating consistent infarct sizes, with n = 2-3 per each MI model and its respective treatment condition. Data are presented as mean ± SEM. Source data are provided as a Source Data file.

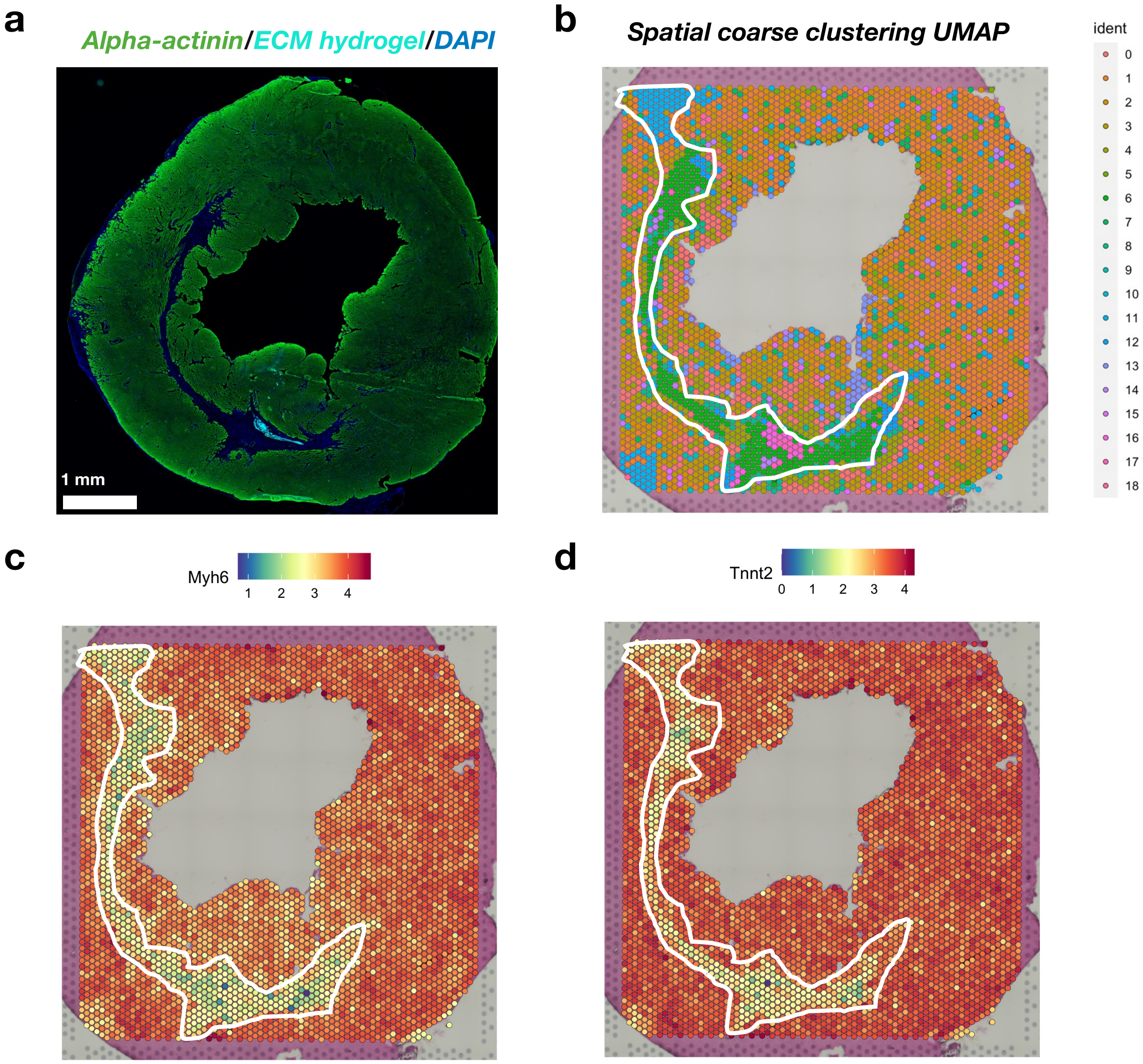

Supplementary Information Figure 2. Strategy for identifying the infarct zone in spatial samples in subacute MI model. (a) Myocardium (green) was labelled with an anti-alpha-actinin antibody, with a white outline indicating the infarct and the ECM hydrogel fluorescently tagged in

light blue. **(b-d)** The adjacent section was used for 10X Visium, with coarse clustering populations identified **(b)**. *Myh6* **(c)** and *Tnnt2* **(d)**, two markers for healthy myocardium, were used to segment and identify clusters that are *Myh6* and *Tnnt2* low, with white outlines overlayed onto the coarse clustering plot, indicating which clusters are infarct specific. Source data are provided as a Source Data file.

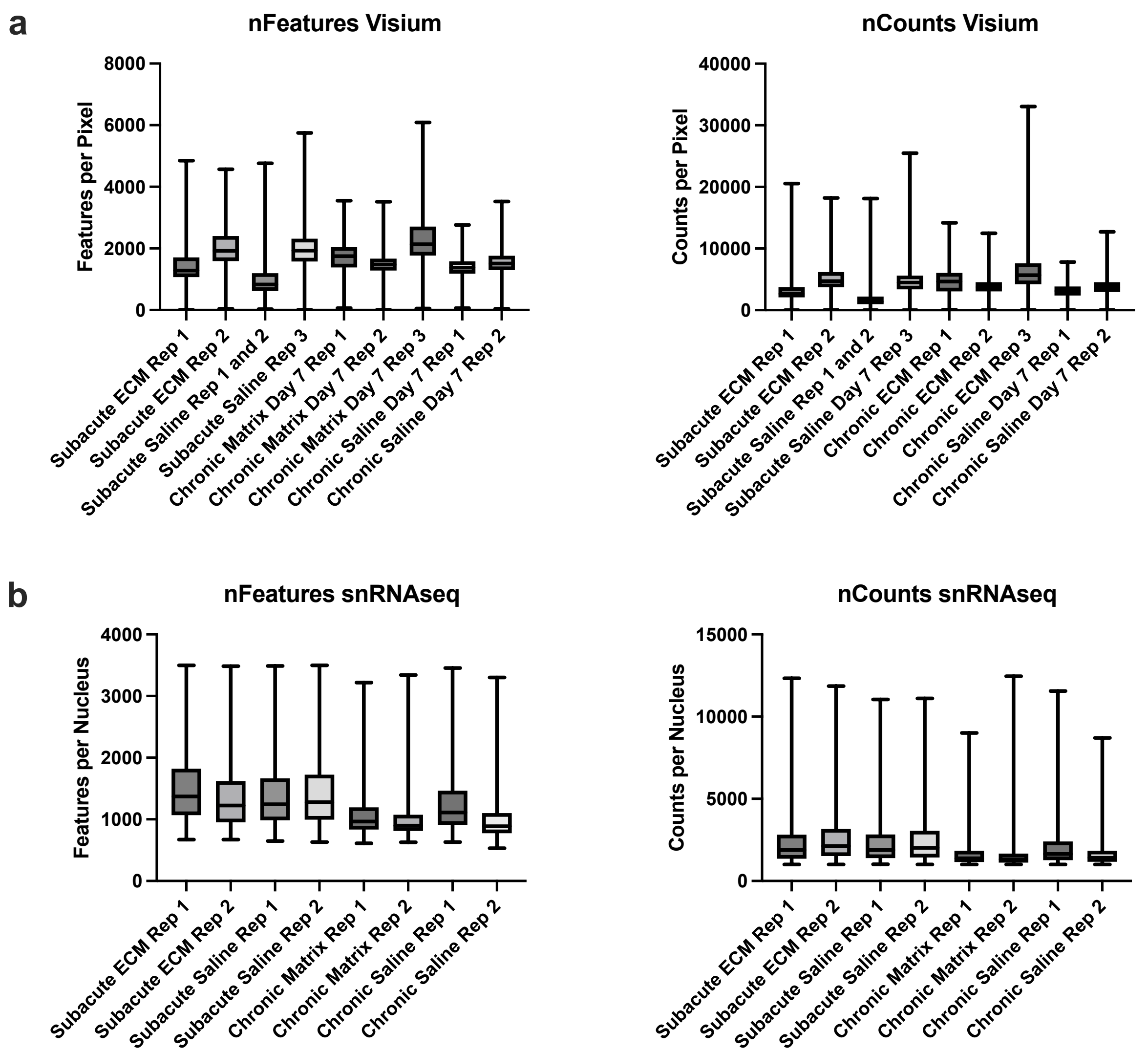

Supplementary Information Figure 3. Transcriptomic quality control for Visium and snRNAseq samples. (a) Quality metrics of samples and replicates for Visium samples

represented in features per pixel (nFeatures) and genes per sample (nCounts). Data are presented as box and whisker plots. **(b)** Quality metrics of samples and replicates for snRNAseq samples represented in features per nuclei (nFeatures) and genes per nuclei (nCounts). Data are presented as box and whisker plots. Source data are provided as a Source Data file.

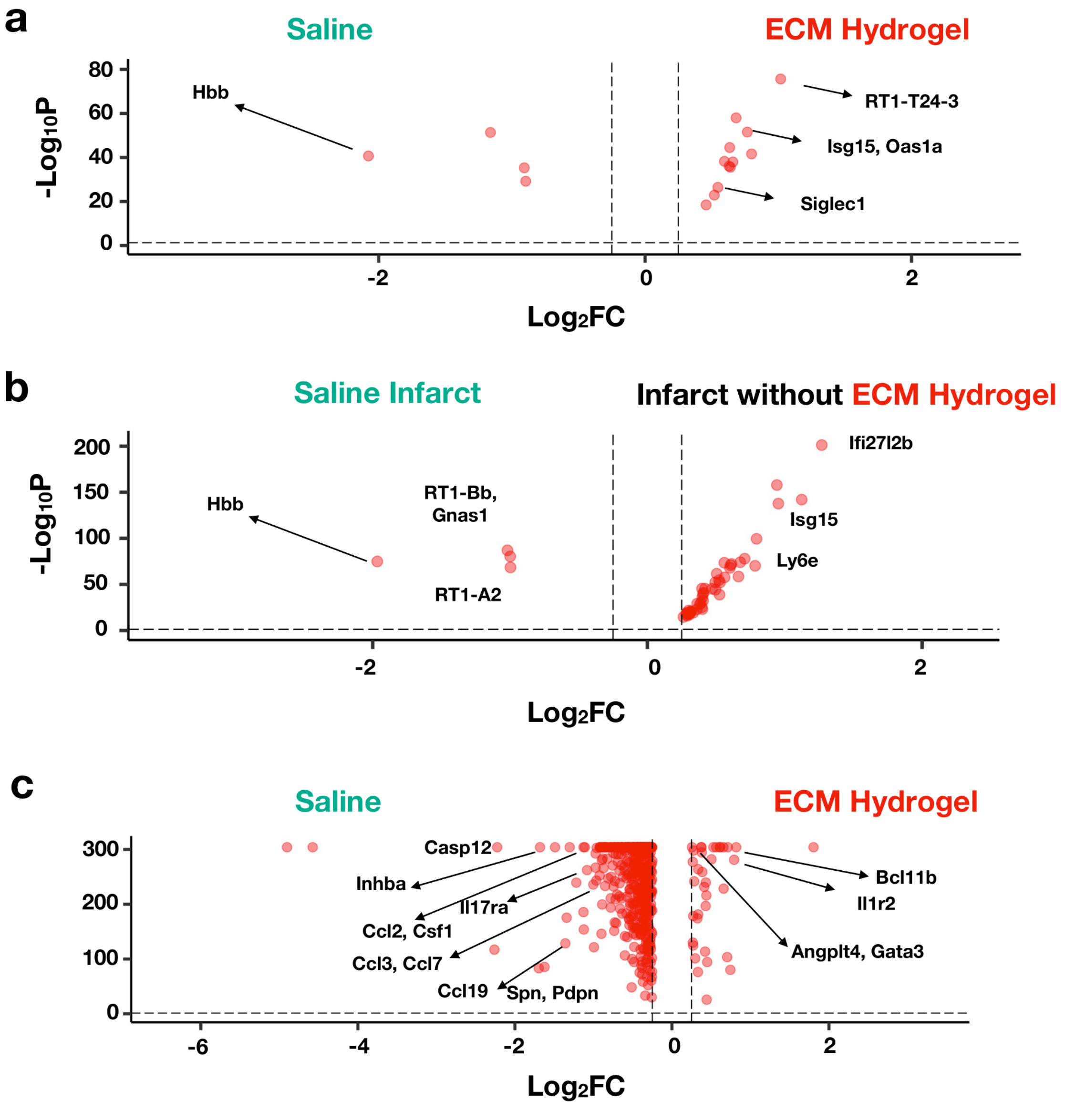

Supplementary Information Figure 4. Further subacute Visium phenotypic differences between ECM hydrogel and saline treatment. (a) Top differentially expressed genes for the

subacute MI model. **(b)** Comparison of infarcts between conditions, where spatial transcriptomic data of ECM hydrogel treated infarcts without fluorescent ECM is compared to saline infarcts. **(c)** Top differentially expressed genes for the remote zones of ECM hydrogel treated spatial samples vs. the remote zones of saline treated samples. Sample size: n = 2 subacute ECM hydrogel (7658 spots), n = 3 subacute saline (8036 spots). Significance was determined via nonparametric Wilcoxon rank-sum tests with a Benjamini–Hochberg FDR adjustment to determine gene lists. Source data are provided as a Source Data file.

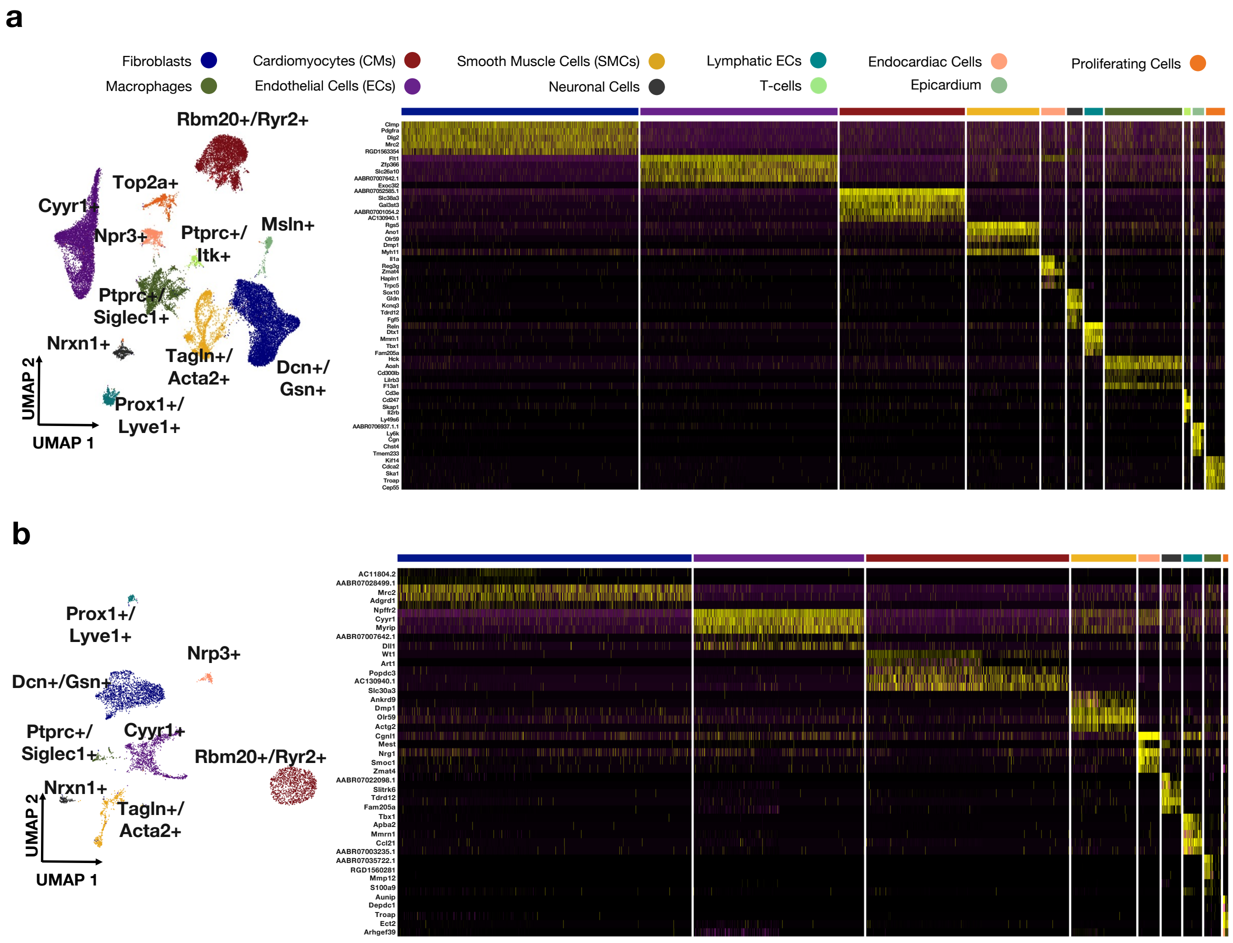

Supplementary Information Figure 5. Feature subsetting and top genes for canonical cell types. (a) Gene subsetting strategy overlayed onto UMAP for subacute cell types, alongside heatmap of top 5 genes per each cell type. **(b)** Gene subsetting strategy overlayed onto UMAP for chronic cell types, alongside heatmap of top 5 genes per each cell type. Source data are provided as a Source Data file.

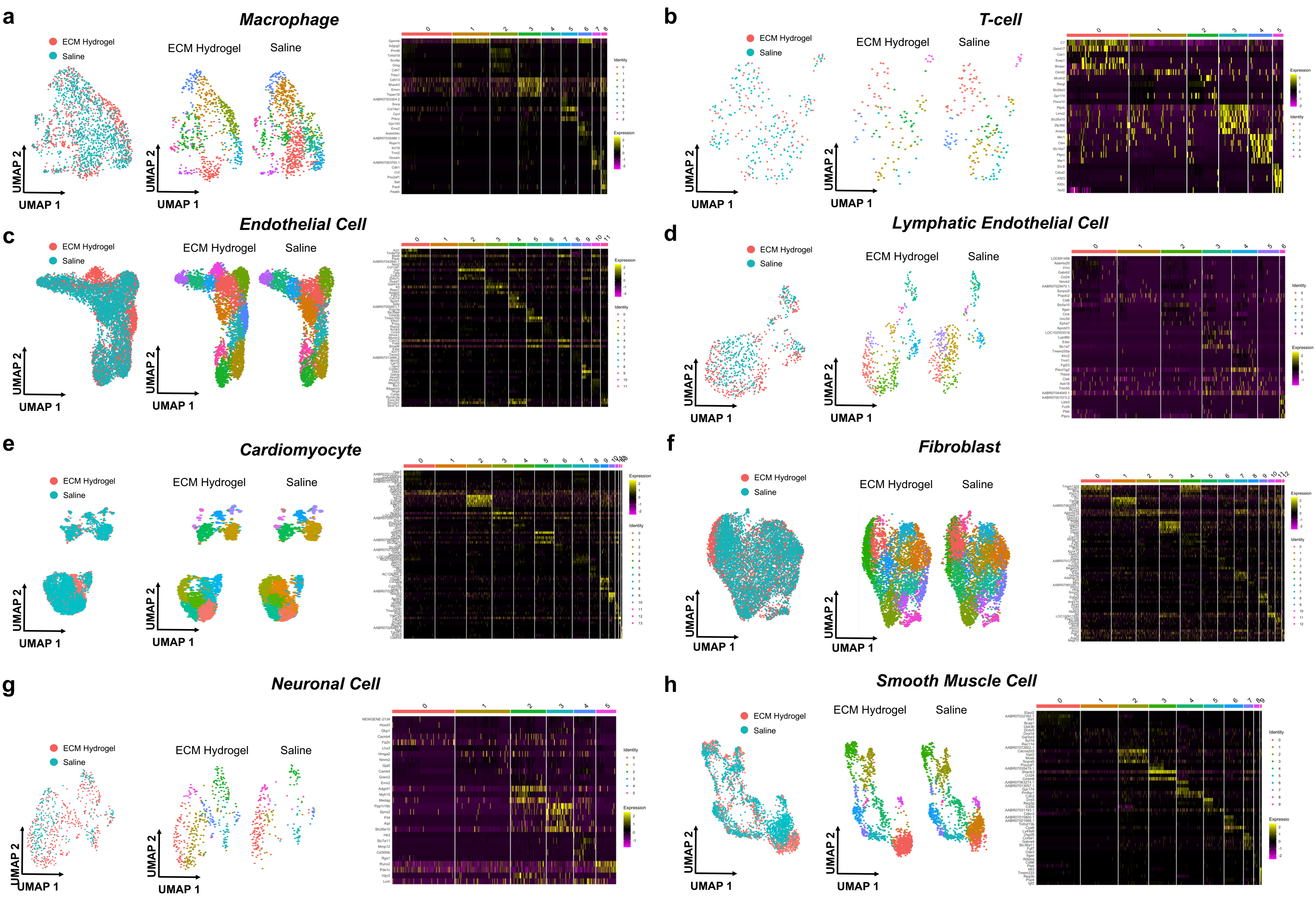
a
Macrophage
ECM Hydrogel
Saline
UMAP 1
UMAP 2
Identity
Expression
b
T-cell
ECM Hydrogel
Saline
UMAP 1
UMAP 2
c
Endothelial Cell
ECM Hydrogel
Saline
UMAP 1
UMAP 2
d
Lymphatic Endothelial Cell
ECM Hydrogel
Saline
UMAP 1
UMAP 2
e
Cardiomyocyte
ECM Hydrogel
Saline
UMAP 1
UMAP 2
f
Fibroblast
ECM Hydrogel
Saline
UMAP 1
UMAP 2
g
Neuronal Cell
ECM Hydrogel
Saline
UMAP 1
UMAP 2
h
Smooth Muscle Cell
ECM Hydrogel
Saline
UMAP 1
UMAP 2

Supplementary Information Figure 6. Subclusters of subsetted cell types in subacute MI.

(a) Macrophages, **(b)** T-cells**,** **(c)** endothelial cells, **(d)** lymphatic endothelial cells, **(e)** cardiomyocytes, **(f)** fibroblasts, **(g)** neuronal cells, and **(h)** smooth muscle cells were subsetted and reintegrated. They were then reclustered at resolution 1, with the top 5 marker genes for each subcluster. Source data are provided as a Source Data file.

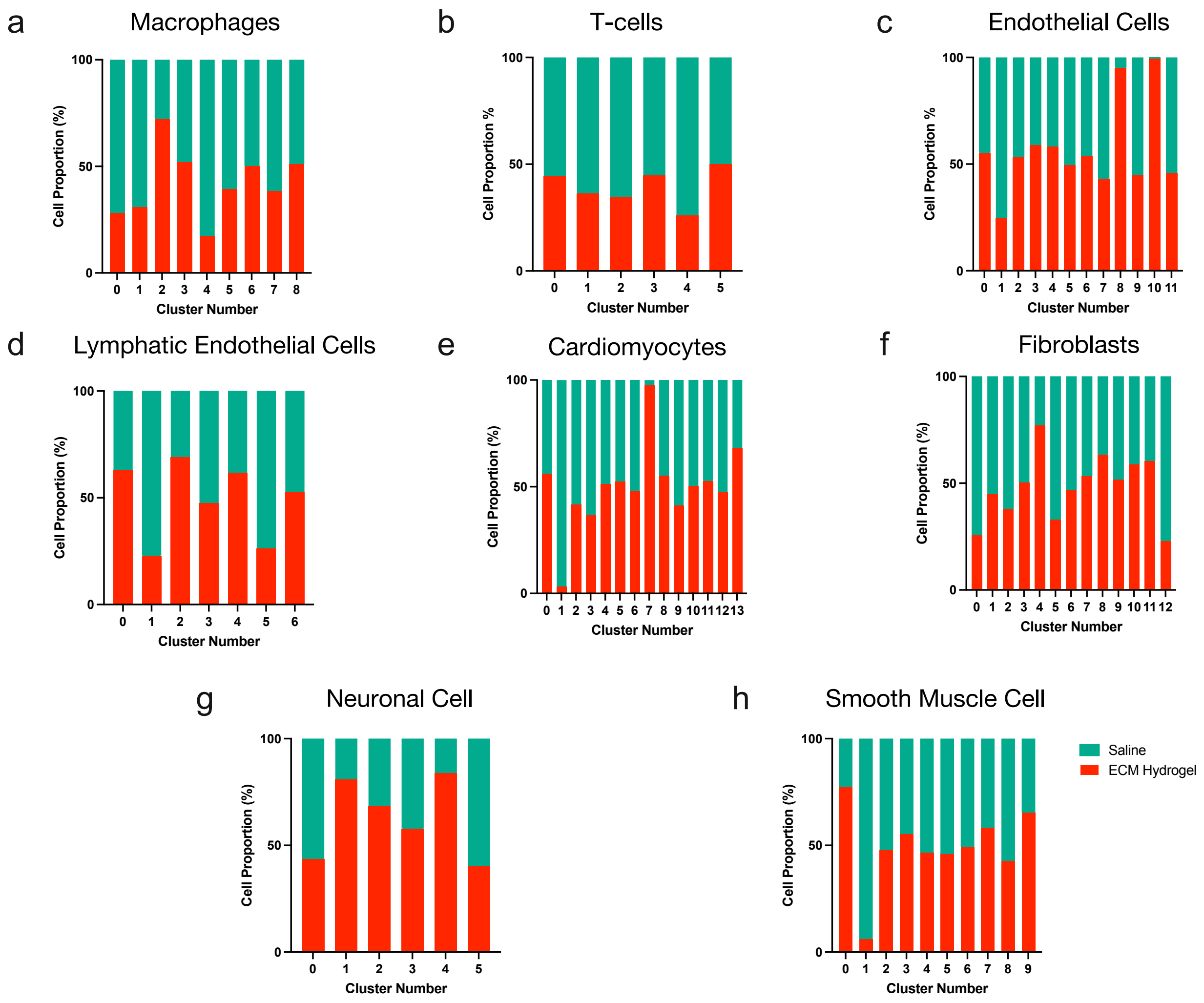

Supplementary Information Figure 7. Cell proportions by treatment of subsetted cell types in subacute myocardial infarction. (a-h) Average subcluster composition of ECM hydrogel and saline for subsetted macrophages **(a)**, T-cells **(b)**, endothelial cells **(c)**, lymphatic endothelial cells **(d)**, cardiomyocytes **(e)**, fibroblasts **(f)**, neuronal cells **(g)**, and smooth muscle cells **(h)**. Source data are provided as a Source Data file.

a

Saline Macrophage Enrichment

GO Term	Adj. p-val
Reg. of cell activation	4.9e-05
Reg. of immune response	2.5e-04
Cell adhesion	4.4e-04
Phagocytosis	5.4e-04
Pos. reg. of immune system process	5.4e-04
Inflammatory response	7.9e-04
Defense response	1.1e-03

b

Saline Fibroblast Enrichment

GO Term	Adj. p-val
Aerobic electron transport chain	4.1e-03
Collagen containing ECM	7.5e-03
Oxidative phosphorylation	1.3e-02
External encapsulating structure	1.6e-02
Aerobic respiration	2.1e-02
Cellular respiration	4.3e-02
Cell adhesion	4.9e-02

Supplementary Information Figure 8. Saline GO terms in subacute MI model. (a) Macrophage enrichment of genes upregulated in saline treatment for the subacute MI model are shown. **(b)** Fibroblast enrichment of genes upregulated in saline treatment for subacute MI model are shown. Significance was calculated using the gene set enrichment analysis software via Kolmogoro-Smirnov tests and permutation testing, with Benjamin-Hochberg FDR adjustment. Source data are provided as a Source Data file. Abbreviations: ECM: extracellular matrix; EC: endothelial cell; CM: cardiomyocyte: SMC: smooth muscle cell; UMAP: uniform manifold approximation and projection; reg: regulation; Pop: population; Prolif: proliferation.

a

T-cells

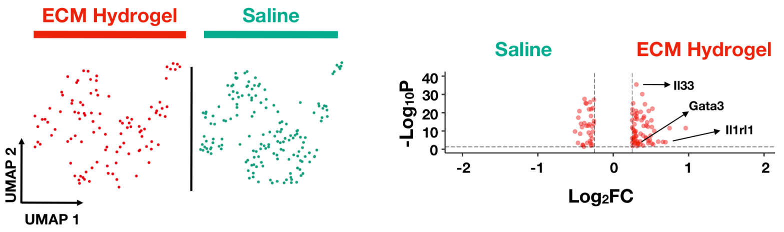

ECM Hydrogel Enrichment

GO Term	Adj. p-val
Cell adhesion	6.9e-07
Neg. reg. of cytokine production	1.3e-05
Heparin binding	9.8e-05
Neg. reg. of type II interferon production	1.3e-04
Pos. reg. of IL-5 production	2.2e-04
Glycosaminoglycan binding	3.4e-04
Reg. of response to stress	5.8e-04

Saline Enrichment

GO Term	Adj. p-val
Neg. reg. of gene expression	7.3e-03
Cell adhesion	1.0e-02
Neg. reg, of cytoplasmic translation	1.5e-02
Transporter activity	1.5e-02
Monoatomic ion transport	1.6e-02
Reg. off transport	2.8e-02
Export from cell	3.7e-02

b

Lymphatic ECs

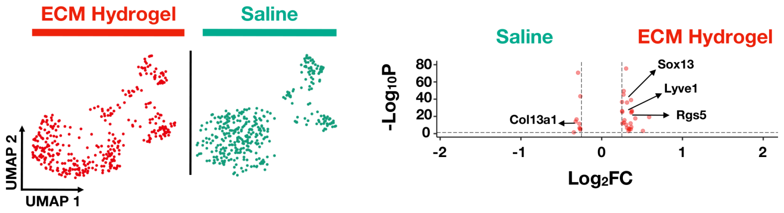

ECM Hydrogel Enrichment

GO Term	Adj. p-val
Tube morphogenesis	2.7e-04
Cell motility	8.9e-04
Circulatory system development	3.9e-03
MAPK cascade	4.4e-03
Vasculature development	2.6e-02
Locomotion	3.6e-02
Cell-cell junction	4.8e-02

Saline Enrichment

GO Term	Adj. p-val
N/A	N/A

c

Neuronal Cells

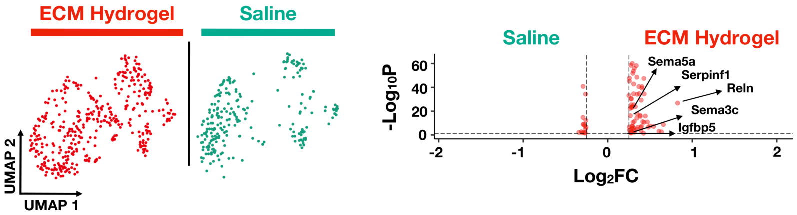

ECM Hydrogel Enrichment

GO Term	Adj. p-val
Cell motility	4.7e-09
Actin binding	5.5e-06
Reg. of neurogenesis	7.2e-05
Cell projection organization	1.1e-04
Somatodendritic compartment	1.3e-04
Reg. of cell projection organization	2.5e-04
Neuron generation	2.5e-04

Saline Enrichment

GO Term	Adj. p-val
N/A	N/A

d

Smooth Muscle Cells

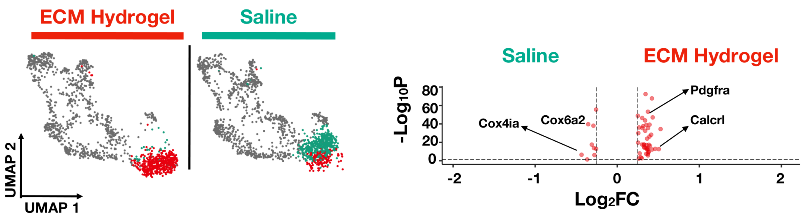

ECM Hydrogel Enrichment

GO Term	Adj. p-val
Reg. of locomotion	8.7e-09
Heart development	1.8e-05
Homeostatic process	1.9e-05
Circulatory system development	1.9e-05
Smooth muscle cell proliferation	3.1e-05
Muscle structure development	2.1e-04
Angiogenesis	2.9e-04

Saline Enrichment

GO Term	Adj. p-val
Inner mito. membrane protein complex	2.8e-02
Mitochondrial electron transport	2.8e-02
Mitochondrial envelope	3.0e-02
Respiratory chain complex IV	3.1e-02
Mitochondrial protein containing complex	3.6e-02
Cytochrome complex	4.4e-02

Supplementary Information Figure 9. Therapeutic differences in other cell types in subacute MI model. (a-d) T-cell **(a)**, lymphatic endothelial cells (EC) **(b)**, neural cells **(c)** and smooth muscle cells **(d)** were subsetted and reclustered into UMAP space. For each cell type, unique clusters to both ECM hydrogel and saline were compared, with their differentially expressed genes displayed in a Volcano plot. All ECM hydrogel specific differentially expressed genes were subjected to GO enrichment. Sample size: n = 2 replicates of subacute ECM hydrogel (22230 cells); n = 2 replicates of subacute saline (18537 cells). Significance was determined via nonparametric Wilcoxon rank-sum tests with a Benjamini–Hochberg FDR adjustment to determine gene lists and via Kolmogoro-Smirnov tests and permutation testing, with Benjamin-Hochberg FDR adjustment **(a-d)**. Source data are provided as a Source Data file. Abbreviations: ECM: extracellular matrix; EC: endothelial cell; CM: cardiomyocyte: SMC: smooth muscle cell; UMAP: uniform manifold approximation and projection; reg: regulation; Pop: population; Prolif: proliferation; Dev: development, FC: fold change.

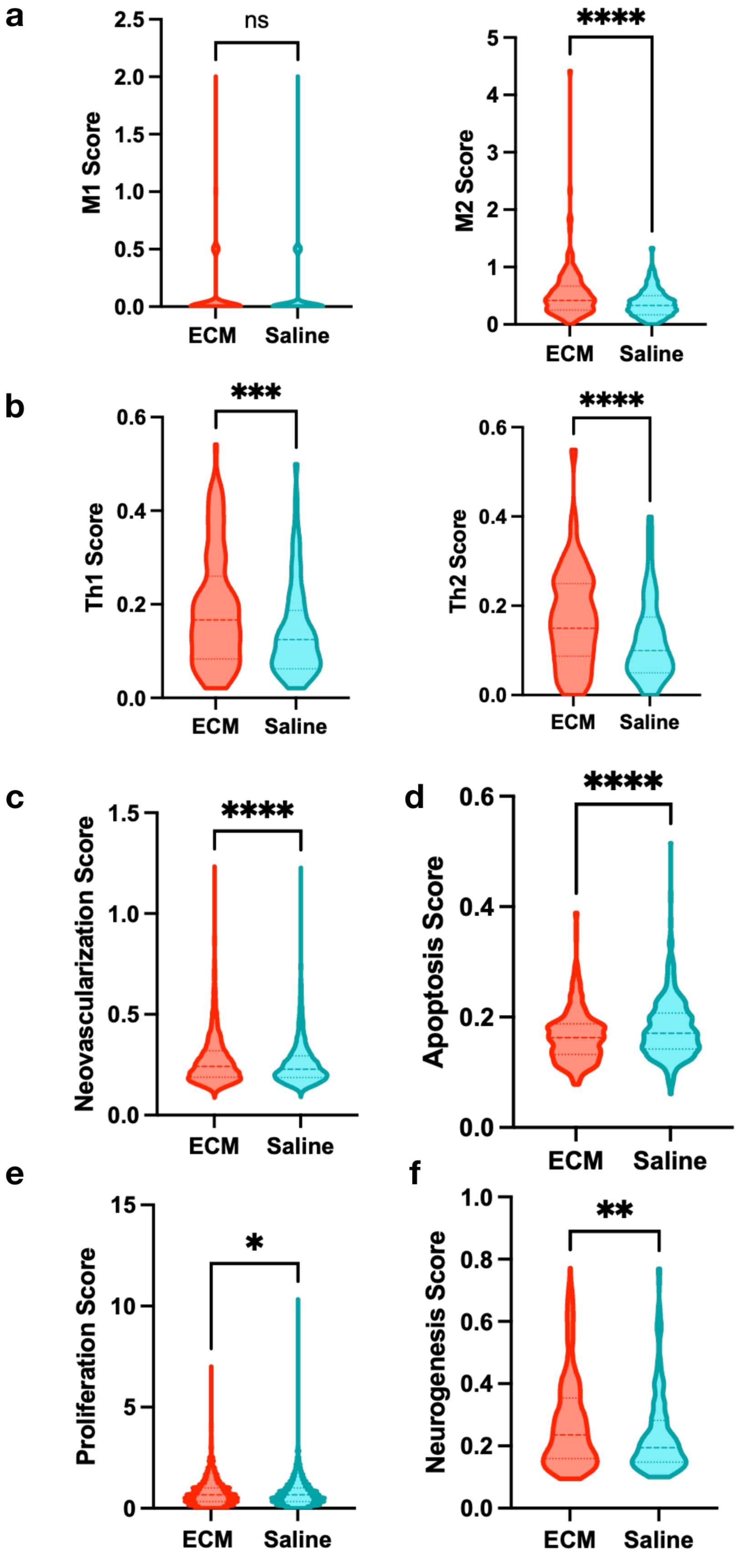
a
ns
M1 Score
2.5
2.0
1.5
1.0
0.5
0.0
ECM
Saline
****
M2 Score
5
4
3
2
1
0
ECM
Saline
b
***
Th1 Score
0.6
0.4
0.2
0.0
ECM
Saline
****
Th2 Score
0.6
0.4
0.2
0.0
ECM
Saline
c
****
Neovascularization Score
1.5
1.0
0.5
0.0
ECM
Saline
d
****
Apoptosis Score
0.6
0.4
0.2
0.0
ECM
Saline
e
*
Proliferation Score
15
10
5
0
ECM
Saline
f
**
Neurogenesis Score
1.0
0.8
0.6
0.4
0.2
0.0
ECM
Saline

Supplementary Information Figure 10. Scoring using gene ontology to infer phenotypic differences. **(a-f)** M1 and M2 **(a)**, Th1 and Th2 **(b)**, neovascularization **(c)**, apoptosis **(d)**, proliferation **(e)**, and neurogenesis **(f)** gene ontology genes were scored onto ECM hydrogel and saline treated nuclei. Significance was calculated via a two-tailed Mann-Whitney nonparametric test, with $p < 0.05$, *$p < 0.05$, **$p < 0.01$, ***$p < 0.001$, ****$p < 0.0001$. M1: $t(1745) = 0.625$, $p = 0.5318$, % Confidence Interval [−0.02410, 0.01244]; M2: $t(486) = 5.557$, $p < 0.0001$, % Confidence Interval [−0.2347, −0.1121]; Th1: $t(486) = 5.557$, $p < 0.0001$, % Confidence Interval: [−0.2347, −0.1121]; Th2: $t(266) = 3.427$, $p = 0.0007$, 95% CI [−0.0728, −0.0197]; Neovascularization: $t(8033) = 5.072$, $p < 0.0001$, 95% CI [−0.01944, −0.00860]; Apoptosis: $t(1121) = 4.238$, $p < 0.0001$, 95% CI [0.00714, 0.01946]; Proliferation: $t(5102) = 2.134$, $p = 0.0329$, 95% CI [−0.07569, −0.00320]; Neurogenesis: $t(630) = 3.295$, $p = 0.0010$, 95% CI: p−0.0603, −0.0153].

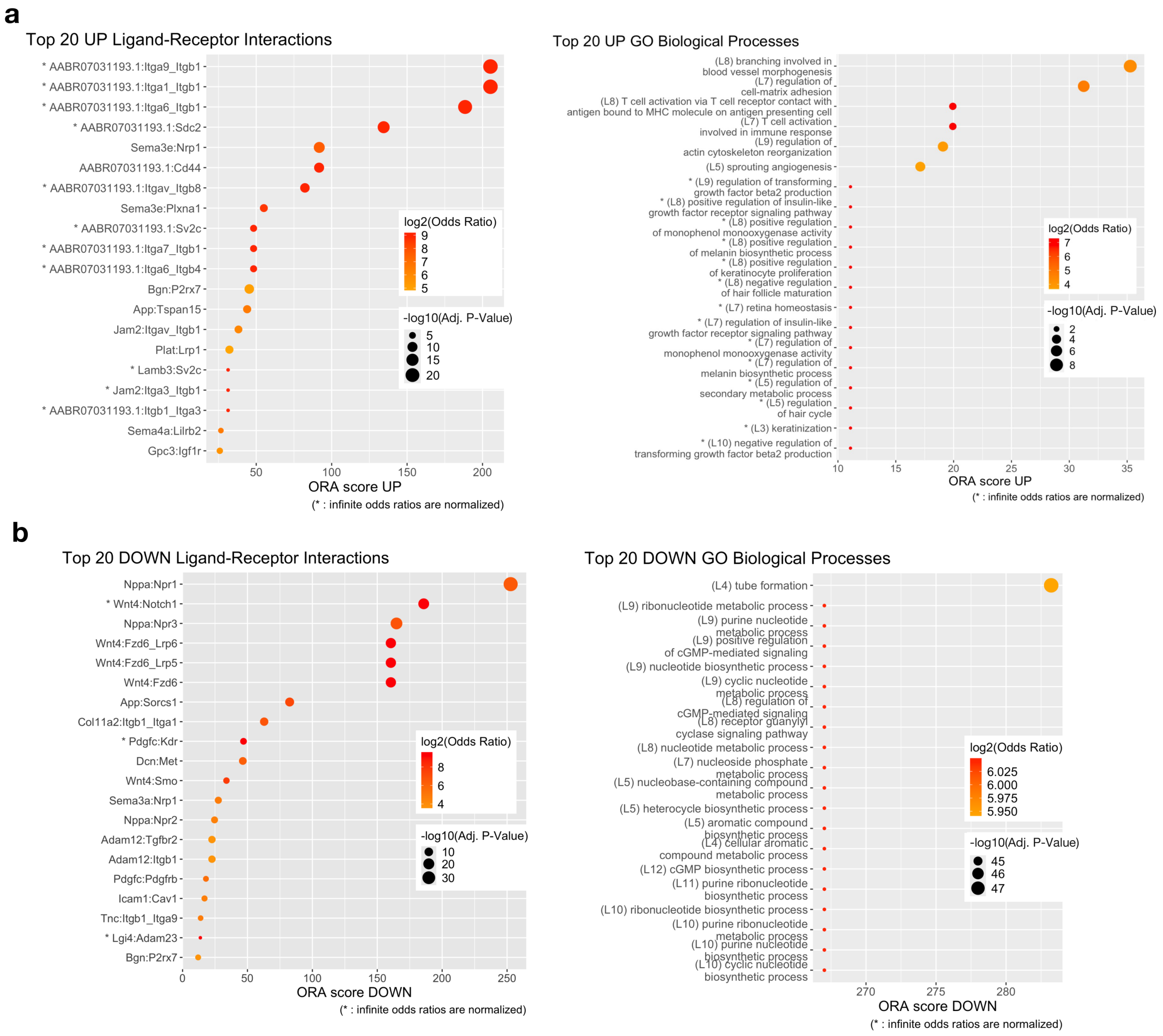

Supplementary Information Figure 11. Ligand receptor ontology for the subacute model. (a) Top upregulated ligand receptor pairings and upregulated GO terms associated with those pairings in the subacute MI model. **(b)** Top downregulated ligand receptor pairings and downregulated GO terms associated with those pairings in the subacute MI model.

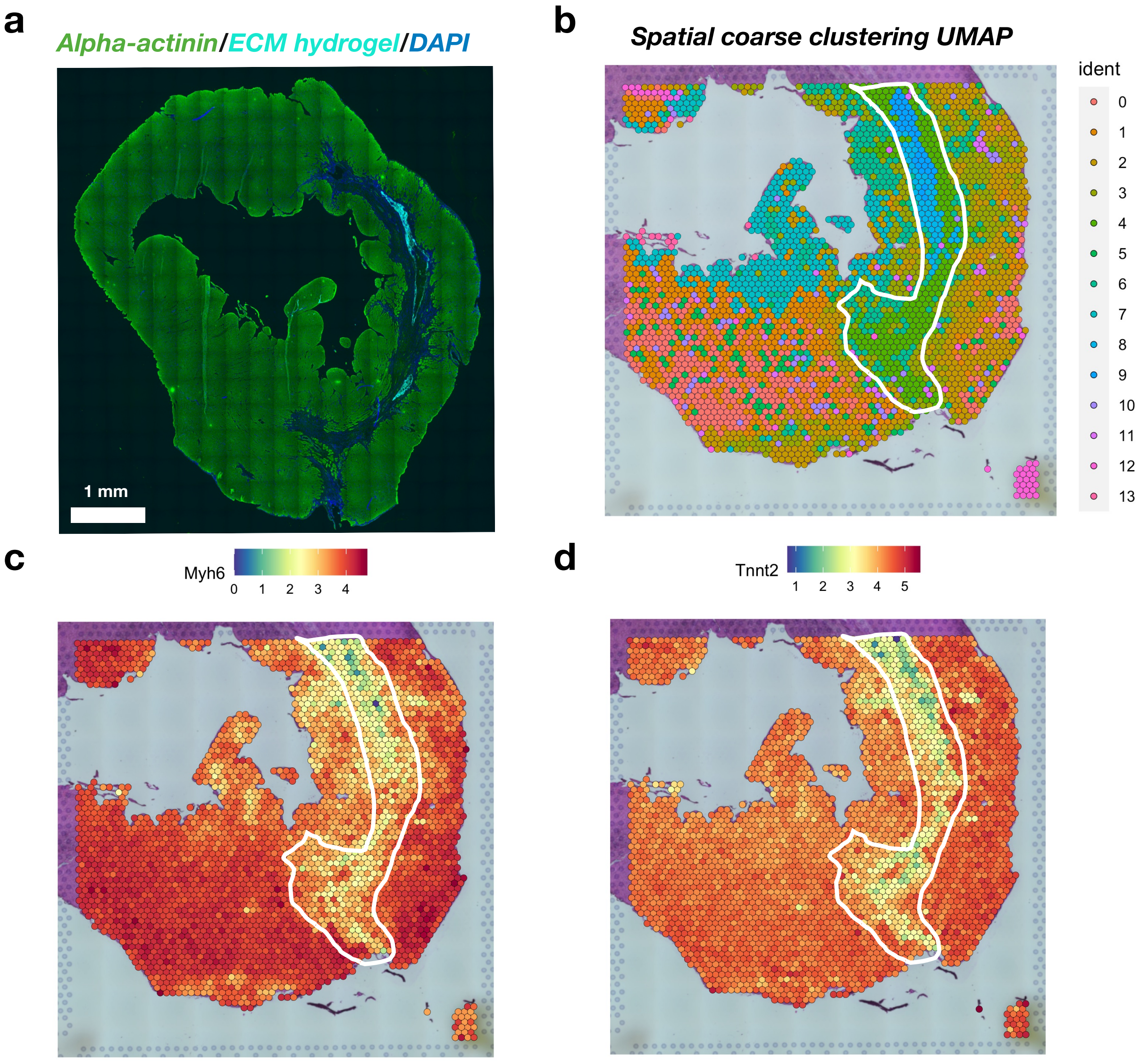

Supplementary Information Figure 12. Strategy for identifying infarct zone in the chronic MI spatial samples. **(a)** Myocardium (green) was labelled with an anti-alpha-actinin antibody, with a white outline indicating the infarct and the ECM hydrogel fluorescently tagged in light blue. **(b-d)** The adjacent section was used for 10X Visium, with coarse clustering populations identified **(b)**. *Myh6* **(c)** and *Tnnt2* **(d)**, two markers for healthy myocardium, were used to segment and identify clusters that are *Myh6* and *Tnnt2* low, with their white outlines overlayed onto the coarse clustering plot, indicating which clusters are infarct specific.

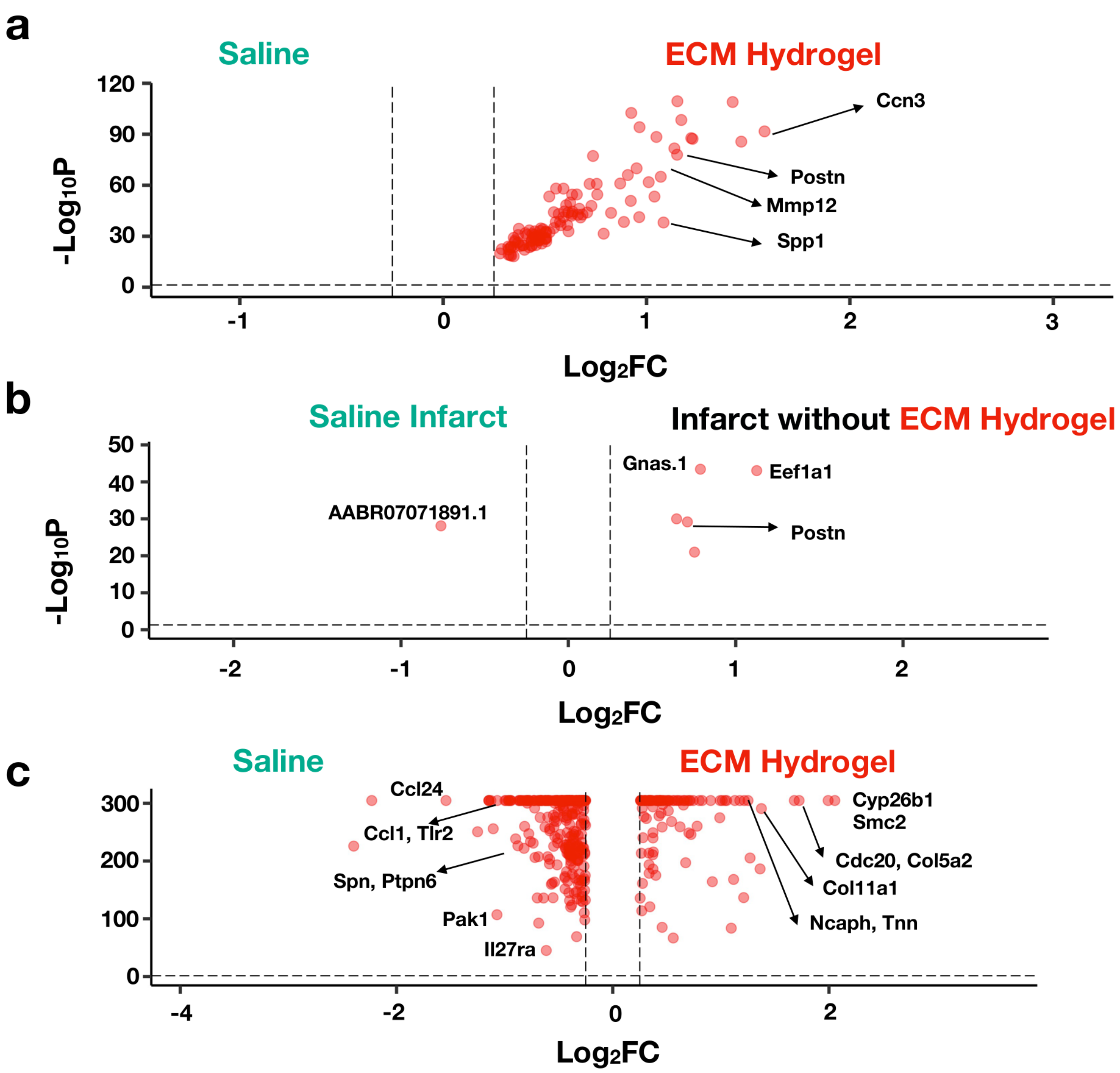

Supplementary Information Figure 13. Further chronic Visium phenotypic differences between ECM hydrogel and saline treatment. (a) Top differentially expressed genes for the chronic MI model. **(b)** Comparison of infarcts between conditions, where spatial transcriptomic data of ECM hydrogel treated infarcts without fluorescent ECM is compared to saline infarcts. **(c)** Top differentially expressed genes for the remote zones of ECM hydrogel treated spatial

samples vs. the remote zones of saline treated samples. Sample size: n = 3 ECM hydrogel replicates, 9594 spots; n = 2 saline replicates, 8166 spots.

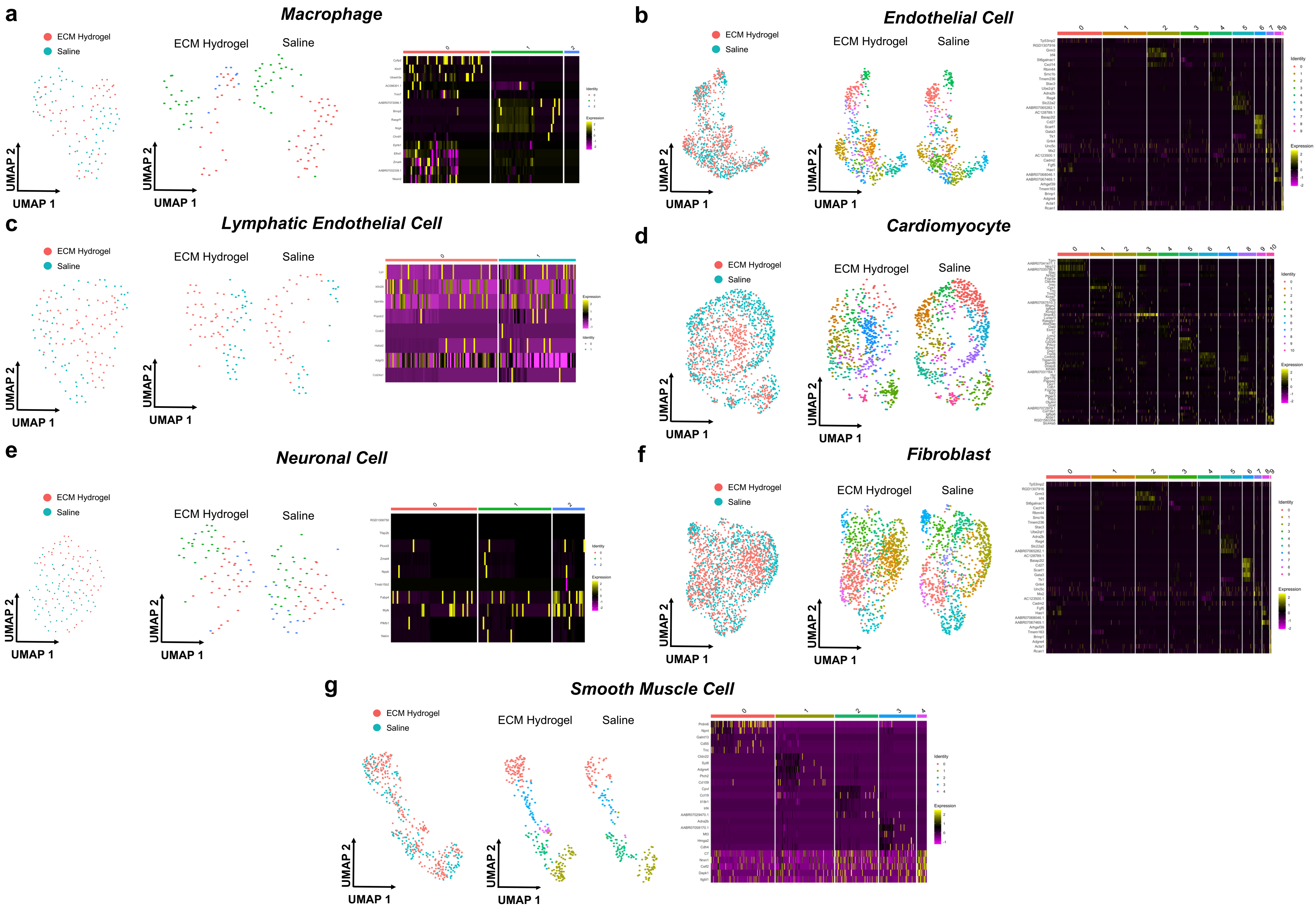
a
Macrophage
ECM Hydrogel
Saline
ECM Hydrogel
Saline
UMAP 1
UMAP 2
b
Endothelial Cell
ECM Hydrogel
Saline
ECM Hydrogel
Saline
UMAP 1
UMAP 2
c
Lymphatic Endothelial Cell
ECM Hydrogel
Saline
ECM Hydrogel
Saline
UMAP 1
UMAP 2
d
Cardiomyocyte
ECM Hydrogel
Saline
ECM Hydrogel
Saline
UMAP 1
UMAP 2
e
Neuronal Cell
ECM Hydrogel
Saline
ECM Hydrogel
Saline
UMAP 1
UMAP 2
f
Fibroblast
ECM Hydrogel
Saline
ECM Hydrogel
Saline
UMAP 1
UMAP 2
g
Smooth Muscle Cell
ECM Hydrogel
Saline
ECM Hydrogel
Saline
UMAP 1
UMAP 2

Supplementary Information Figure 14. Subclusters of subsetted cell types in chronic MI. **(a)** Macrophages, **(b)** endothelial cells, **(c)** lymphatic endothelial cells, **(d)** cardiomyocytes, **(e)** neuronal cells, **(f)** fibroblasts, and **(g)** smooth muscle cells were subsetted and reintegrated. They were then reclustered at resolution 1, with the top 5 marker genes for each subcluster. Source data are provided as a Source Data file.

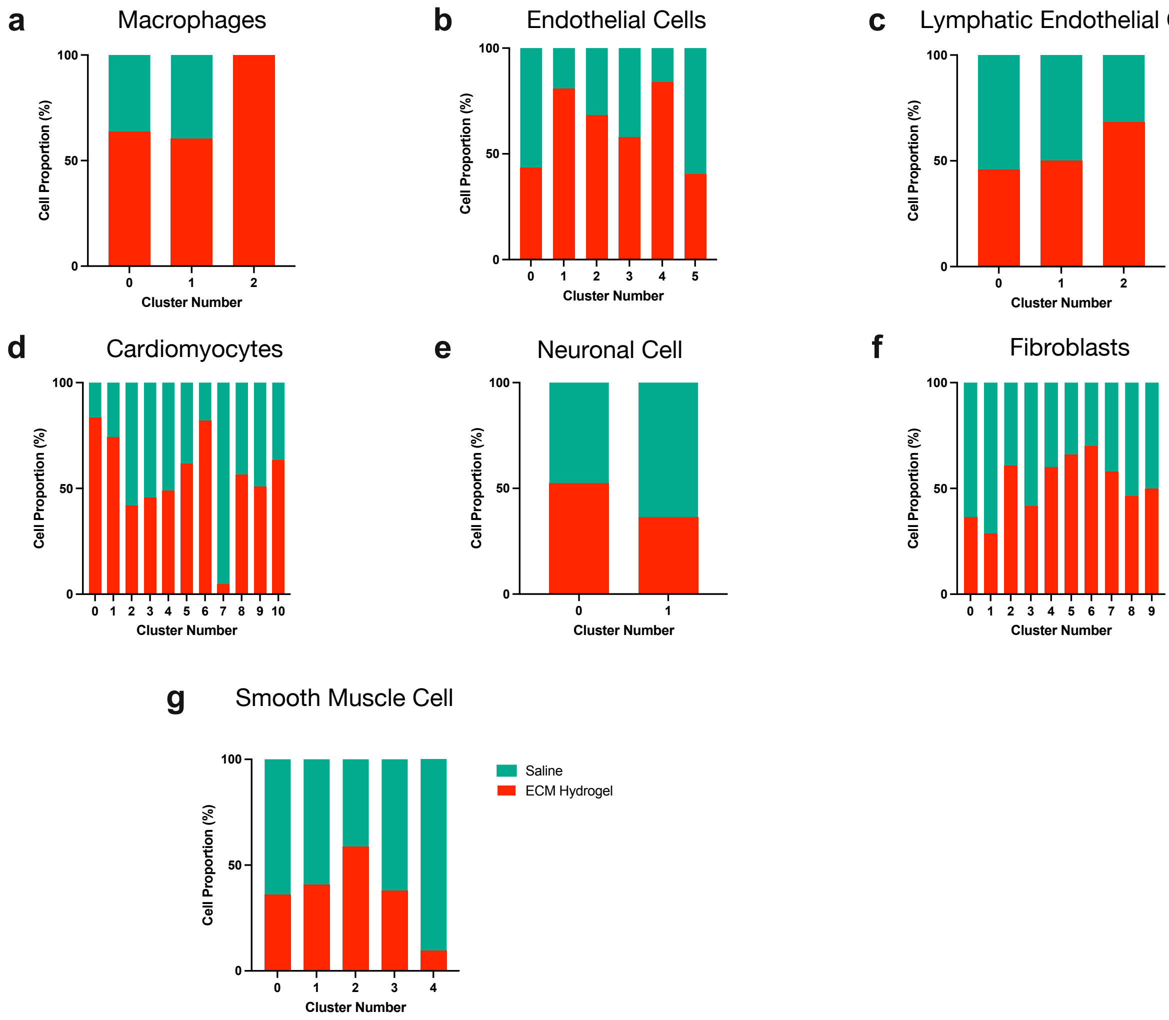

Supplementary Information Figure 15. Cell Proportions by treatment of subsetted cell types in chronic myocardial infarction. (a-h) Average subcluster composition of ECM hydrogel

and saline for subsetted macrophages **(a)**, endothelial cells **(b)**, lymphatic endothelial cells **(c)**, cardiomyocytes **(d)**, neuronal cells **(e)**, fibroblasts **(f)**, and smooth muscle cells **(g)**. Source data are provided as a Source Data file.

a

Lymphatic ECs

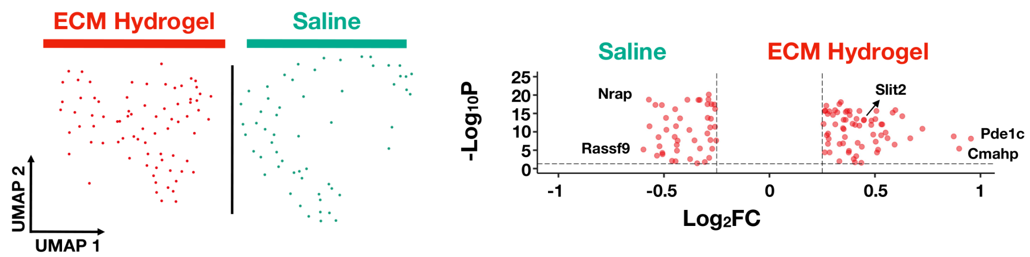

ECM Hydrogel Enrichment

GO Term	Adj. p-val
Tube morphogenesis	2.0e-07
Tube development	1.9e-06
Blood vessel morphogenesis	7.6e-06
Reg. of Wnt signaling pathway	4.3e-03
Circulatory system development	5.3e-03
Angiogenesis	6.3e-03
Vasculature development	7.6e-03

Saline Enrichment

GO Term	Adj. p-val
Cell motility	2.4e-05
Locomotion	1.5e-03
Reg. of cell population proliferation	1.7e-03
Neg. reg of response to stimulus	5.8e-03
Neg. reg. of developmental process	5.8e-03
Pos. reg. of leukocyte cell-cell adhesion	5.8e-03
Actin filament-based process	6.3e-03

b

Neuronal Cells

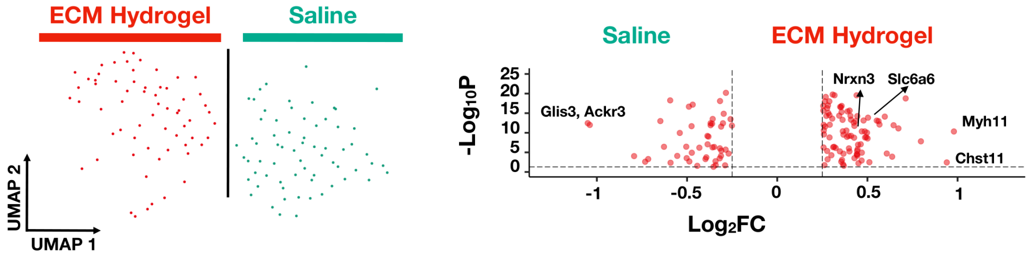

ECM Hydrogel Enrichment

GO Term	Adj. p-val
Neuron projection	3.9e-11
Dendritic tree	1.3e-11
Postsynapse organization	1.4e-09
Neuron development	3.9e-09
Neurogenesis	7.3e-09
Synapse assembly	7.6e-09
Circulatory system development	3.3e-08

Saline Enrichment

GO Term	Adj. p-val
Circulatory system development	1.0e-04
Supramolecular polymer	2.7e-03
Cytoskeletal protein binding	2.7e-03
Muscle organ development	4.1e-03
Neg. reg. of cell communication	4.1e-03
Cardiac muscle contraction	5.1e-03
Neg. reg. of cardiac muscle adaption	1.1e-02

c

Smooth Muscle Cells

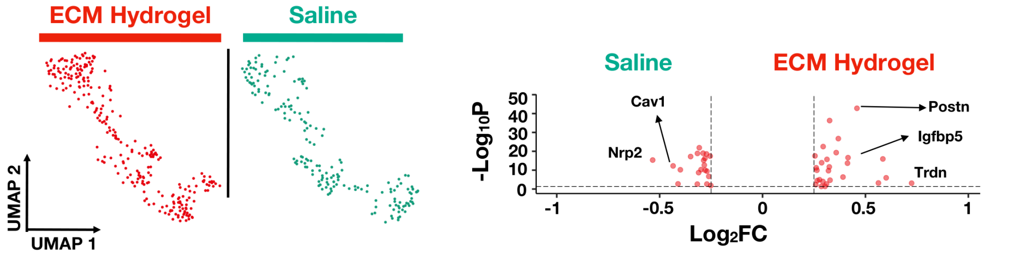

ECM Hydrogel Enrichment

GO Term	Adj. p-val
Reg. of cGMP mediated signaling	1.1e-03
Cytochrome complex	4.2e-03
Heparin binding	5.5e-03
Glycosoaminoglycan binding	1.0e-02
Cell growth	1.2e-02
Pos. reg. of developmental processes	1.6e-02
Muscle hypertrophy	2.4e-02

Saline Enrichment

GO Term	Adj. p-val
Animal organ morphogenesis	5.5e-03
ECM tensile strength	5.5e-03
Homeostatic process	5.5e-03
Vasculature development	5.5e-03
Pos. reg. of cell adhesion	9.4e-03
Angiogenesis	1.2e-02
Collagen trimer	1.3e-03

Supplementary Information Figure 16. Therapeutic differences in other cell types in the chronic model. (a-c) lymphatic endothelial cells (EC) **(a)**, neuronal cells **(b)**, smooth muscle cells **(c)** were subsetted and reclustered into UMAP space. For each cell type, unique clusters to both ECM and saline were compared, with their differentially expressed genes displayed in a Volcano plot. All ECM specific differentially expressed genes were subjected to GO enrichment. Sample size: n = 2 replicates of chronic ECM hydrogel (3160 cells), n = 2 replicates of chronic saline (2952 cells)**.** Source data are provided as a Source Data file. Abbreviations: ECM: extracellular matrix; EC: endothelial cell; CM: cardiomyocyte: SMC: smooth muscle cell; UMAP: uniform manifold approximation and projection; reg: regulation; Pop: population; Prolif: proliferation; Dev: development, FC: fold change.

a Saline Macrophage Enrichment

GO Term	Adj. p-val
Reg. of positive chemotaxis	1.9e-04
Reg. of anatomical structure morph.	3.6e-04
Extracellular matrix binding	1.2e-03
Locomotion	2.3e-03
Chloride transport	3.9e-03
Transmembrane transport	3.9e-03
Taxis	5.7e-03

b Saline Endothelial Cell Enrichment

GO Term	Adj. p-val
Circulatory system development	5.3e-03
Response to metal iron	2.2e-02
Cell-cell signaling	2.8e-02
Regulation of transport	2.8e-02
Heparin sulfate proteoglycan metabolism	2.8e-02
Vasculature development	3.6e-02
Vascular process in circulatory system	3.6e-02

c Saline Cardiomyocyte Enrichment

GO Term	Adj. p-val
Transmembrane transport	8.1e-05
Cytoskeletal protein binding	8.1e-05
Actin filament biding	8.1e-05
Monoatomic ion transmembrane transport	8.1e-05
Actin binding	2.9e-04
Cell morphogenesis	3.7e-04
Transporter activity	6.7e-04

d Saline Fibroblast Enrichment

GO Term	Adj. p-val
Cell adhesion	3.2e-07
Cell projection organization	4.4e-06
External encapsulating structure	4.7e-06
Actin filament based process	1.9e-05
Supramolecular fiber organization	2.4e-05
Cell substrate adhesion	4.0e-05
Collagen containing ECM	6.0e-05

Supplementary Information Figure 17. Saline GO terms in chronic MI model. (a) Macrophage enrichment of genes upregulated in saline treatment for the chronic MI model. **(b)** Endothelial cell enrichment of genes upregulated in saline treatment for the chronic MI model. **(c)** Cardiomyocyte enrichment of genes upregulated in saline treatment for the chronic MI model. **(d)** Fibroblast enrichment of genes upregulated in saline treatment for the chronic MI model. Source data are provided as a Source Data file. Abbreviations: ECM: extracellular matrix; EC: endothelial cell; CM: cardiomyocyte: SMC: smooth muscle cell; UMAP: uniform manifold approximation and projection; reg: regulation; Pop: population; Prolif: proliferation; Morph: morphogenesis; Dev: development, FC: fold change.

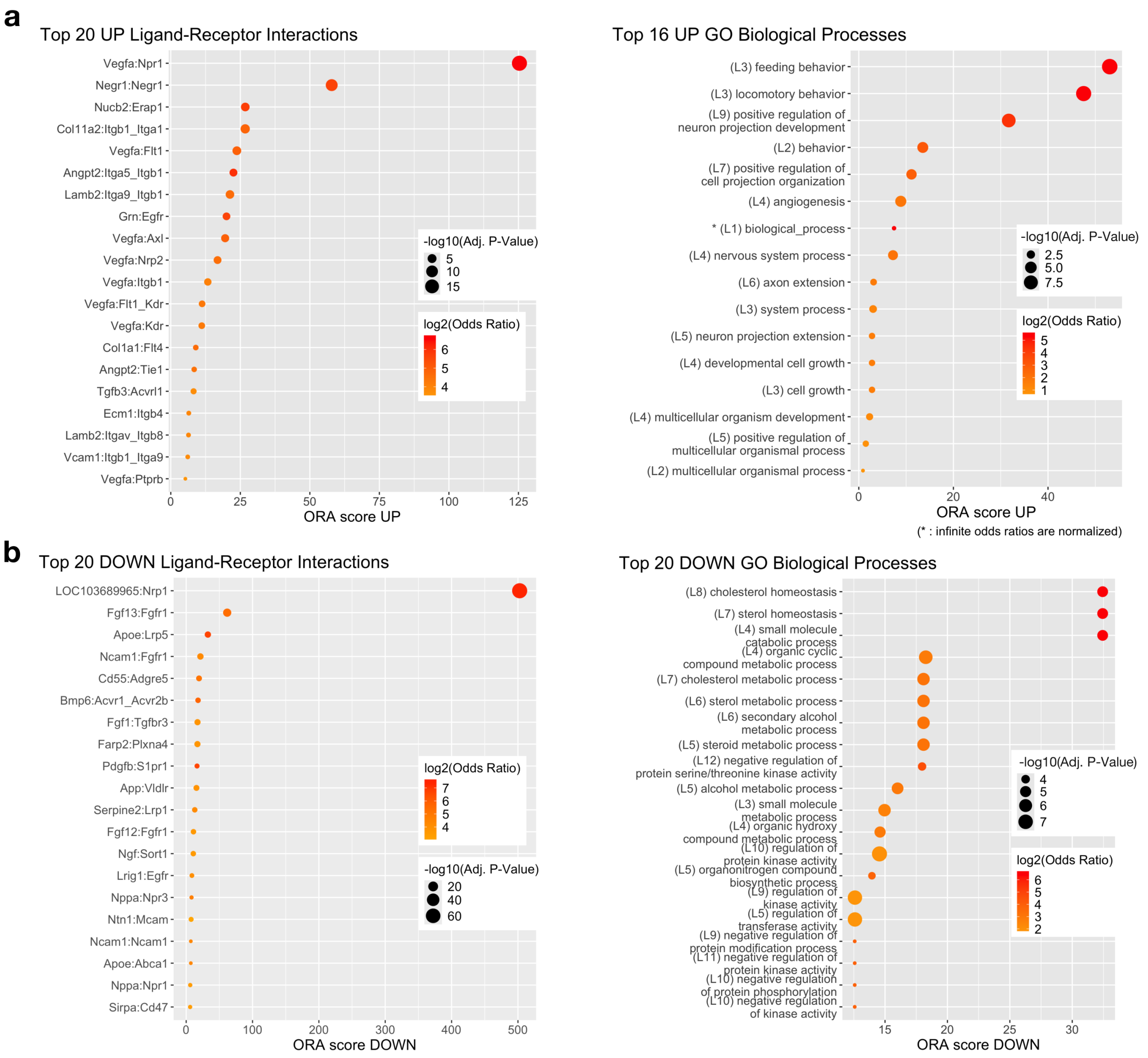

Supplementary Information Figure 18. Ligand receptor ontology for the chronic model. (a) Top upregulated ligand receptor pairings and upregulated GO terms associated with those pairings in the chronic MI model. **(b)** Top downregulated ligand receptor pairings and downregulated GO terms associated with those pairings in the chronic MI model.

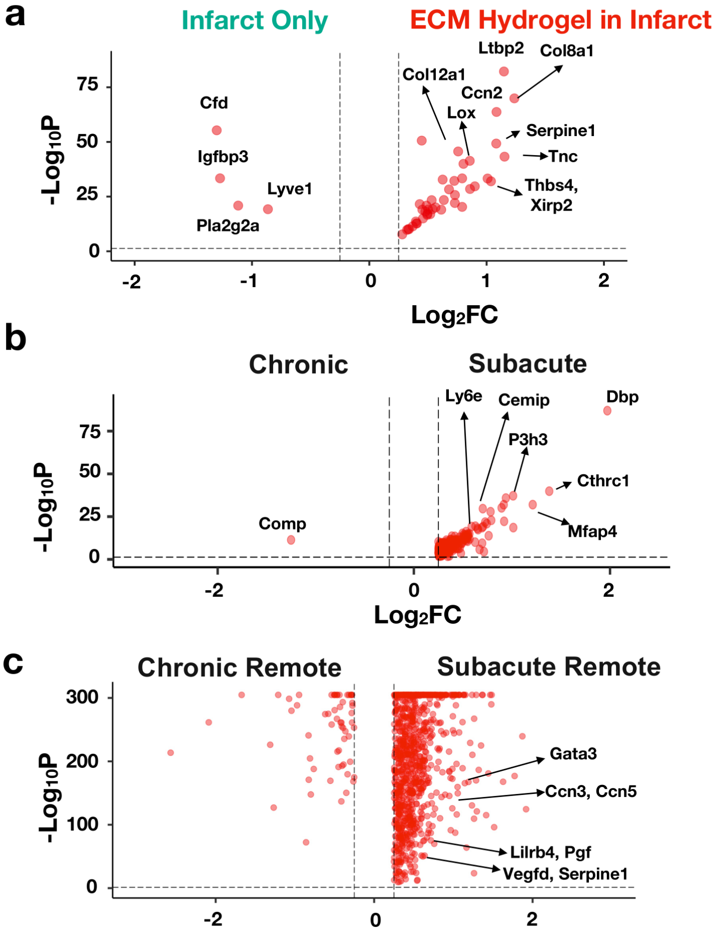

Matrix Zone Enrichment

GO Term	Adj. p-val
External encapsulating structure	1.3e-14
Tube development	9.8e-08
Circulatory system development	1.2e-06
Homeostatic process	7.2e-05
Neuron development	1.7e-04
Leukocyte migration	5.8e-03
Heart development	6.3e-03

Subacute Enrichment

GO Term	Adj. p-val
Circulatory system development	3.4e-17
Tube development	1.2e-16
Neg. reg. of response to stimulus	1.8e-15
Central nervous system development	8.5e-14
Collagen containing ECM	4.5e-12
Regulation of programmed cell death	5.4e-11
Response to wounding	8.5e-10

Supplementary Information Figure 19. Direct Comparison of ECM Hydrogel Administration in Subacute and Chronic Models of MI. (a) Spatial comparison of integrated subacute and chronic MI ECM zones with infarct only zones. The matrix zone's differentially expressed genes were subjected to GO enrichment. **(b)** Direct comparison of spatial subacute and chronic ECM zones, with differentially expressed genes displayed via Volcano Plot. GO enrichment related to the subacute ECM zone are also displayed. **(c)** Top differentially expressed genes for the remote zones of subacute ECM hydrogel treated spatial samples vs. the remote zones of chronic ECM hydrogel treated samples. Sample size: n = 2 subacute ECM hydrogel (7658 spots); n = 3 chronic ECM hydrogel (9594 spots). Source data are provided as a Source Data file. ECM: extracellular matrix; reg: regulation; neg: negative; Resp: response; Org: organization; Pop: population; Prolif: proliferation. FC: fold change.
