# Supplementary material for: Regional and cell specific bioactivity of injectable extracellular matrix biomaterials in myocardial infarction
Source: Nat Commun. 2025 Nov 24;16:10387. doi: 10.1038/s41467-025-65351-5 (PMC12644877; doi:10.1038/s41467-025-65351-5)
Supplement: Supplementary file 2 — Description Of Additional Supplementary File [file 41467_2025_65351_MOESM2_ESM.pdf]

Description of additional supplementary files

File Name: Supplementary Data 1

Description: Differentially Expressed Genes Spatially Comparing ECM Hydrogel in the Infarct and Infarct Only Zones in the Subacute MI Model

File Name: Supplementary Data 2

Description: Global Spatial Comparisons of Infarcts Treated with ECM Hydrogel or Saline in the Subacute MI Model

File Name: Supplementary Data 3

Description: ECM Hydrogel Treated Subacute Infarcts without Visible ECM Compared to Saline Subacute Infarcts

File Name: Supplementary Data 4

Description: Differentially Expressed Genes Spatially Comparing the Remote Zone of ECM Treated Hearts vs. Saline Treated Hearts in a Subacute MI Model

File Name: Supplementary Data 5

Description: Coarse Cluster Markers for Subacute Model

File Name: Supplementary Data 6

Description: Subcluster Markers Genes for Cell Types in Subacute MI

File Name: Supplementary Data 7

Description: Cell-Specific Differentially Expressed Genes with ECM (Up) or Saline (Down) Treatment in the Subacute MI Model

File Name: Supplementary Data 8

Description: Differentially Expressed Genes Spatially Comparing ECM Hydrogel in the Infarct and Infarct Only Zones in the Chronic MI Model

File Name: Supplementary Data 9

Description: Global Spatial Comparisons of Infarcts Treated with ECM Hydrogel or Saline in the Chronic MI Model

File Name: Supplementary Data 10

Description: ECM Hydrogel Treated Chronic Infarcts without Visible ECM Compared to Saline Chronic Infarcts

File Name: Supplementary Data 11

Description: Differentially Expressed Genes Spatially Comparing the Remote Zone of ECM Treated Hearts vs. Saline Treated Hearts in a Chronic MI Model

File Name: Supplementary Data 12

Description: Coarse Cluster Markers for Chronic Model

File Name: Supplementary Data 13

Description: Subcluster Marker Genes in Chronic MI Model

File Name: Supplementary Data 14

Description: Cell-Specific Differentially Expressed Genes with ECM (Up) or Saline (Down) Treatment in the Chronic MI Model

File Name: Supplementary Data 15

Description: Differentially Expressed Genes Spatially Comparing ECM Hydrogel in the Infarct and Infarct Only Zones with Integrated Subacute and Chronic MI

File Name: Supplementary Data 16

Description: Differentially Expressed Genes Spatially Comparing ECM Hydrogel in the Infarct between Subacute and Chronic MI

File Name: Supplementary Data 17

Description: ECM Hydrogel Treated Subacute Remote Zones Vs. ECM Hydrogel Treated Chronic Remote Zones

File Name: Supplementary Data 18

Description: Cell-Specific Differentially Expressed Genes in the Subacute (Up) or Chronic (Down) with ECM Hydrogel Administration
